# Third-Degree Atrioventricular Block Secondary to Lyme Disease: A Case Report

**DOI:** 10.7759/cureus.49803

**Published:** 2023-12-01

**Authors:** Morgan C Ivey, Mohammad Kooshkabadi

**Affiliations:** 1 Medicine, Philadelphia College of Osteopathic Medicine, Moultrie, USA; 2 Cardiology, Wellstar Cardiology Group, Atlanta, USA

**Keywords:** temporary pacemaker, erythema migrans, ixodes ticks, carditis, atrioventricular heart block, lyme's disease

## Abstract

Lyme carditis* *is a disseminated infection caused by the bite of an *Ixodes* tick with the transfer of *Borrelia burgdorferi*. It is characterized by flu-like symptoms, syncope, palpitations, and at times, erythema migrans. It is a rare systemic complication of Lyme disease that occurs when the initial infection is not promptly and completely treated. It is usually seen in patients who spend time outdoors and are thus more likely to get bitten by a tick, though anyone could be affected. This manuscript reports a case of Lyme carditis in a 26-year-old Caucasian male who suffered from flu-like symptoms, syncope, erythema migrans, and third-degree atrioventricular block after a known tick bite. The patient recovered with temporary pacing, doxycycline, and ceftriaxone. Having clinical suspicion of Lyme disease allows for early diagnosis and treatment, thus preventing more serious and systemic complications.

## Introduction

Lyme disease is caused by an infection with a spirochete, Borrelia burgdorferi, that is transmitted via the Ixodes tick. It is the most common tick-borne illness in North America. It is more common in the Northeast and upper Midwest; however, cases have been reported in every state across North America and many countries worldwide [1].

Early localized Lyme disease usually presents with an erythema migrans rash within a month of the tick bite. Lyme disease may disseminate over weeks, presenting with a spreading rash, fatigue, fever, malaise, myalgia, and arthralgia. Without diagnosis and treatment, further dissemination and multi-system involvement may occur, including the nervous system, skin, joints, heart, and eyes [1]. A subset of patients will continue to experience symptoms for significant periods of time, even following treatment. Reported lasting symptoms include fatigue, headache, musculoskeletal pain, cognitive difficulty, paresthesia, dizziness, etc. Having more severe symptoms at diagnosis and having a delay in treatment are risk factors for developing long-term symptoms of Lyme disease. There is currently no FDA-approved treatment for symptoms that persist after completing the recommended course of antibiotics for Lyme disease [2].

Lyme carditis is rare, occurring in approximately 1% of all Lyme disease cases. Infection of the cardiac muscle leads to inflammation and the production of cross-reactive antibodies that disrupt the atrioventricular node, resulting in a high-degree atrioventricular block in 70%-80% of patients with Lyme carditis [3]. This case represents an occurrence of third-degree atrioventricular block secondary to Lyme disease.

## Case presentation

**Figure 1:**
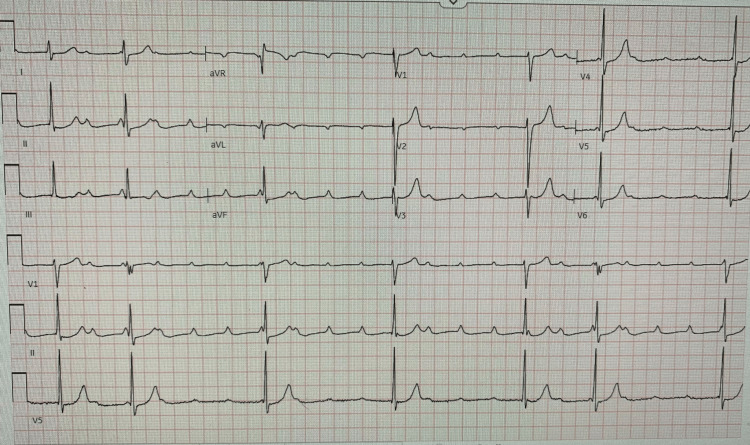
EKG showing third-degree atrioventricular block.

A 26-year-old Caucasian male presented to the emergency department via emergency medical services (EMS) with three episodes of syncope overnight. EMS reported that he was hypotensive, bradycardic at around 20 beats per minute, and was exhibiting third-degree atrioventricular heart block (Figure 1). The patient reported a period of mild cough and congestion that occurred a few weeks prior and had since resolved. He also had experienced the sensation of his heart beating in his throat for a few days prior to admission. Most importantly, he had a tick bite one month earlier while visiting his family in Massachusetts.

**Figure 2:**
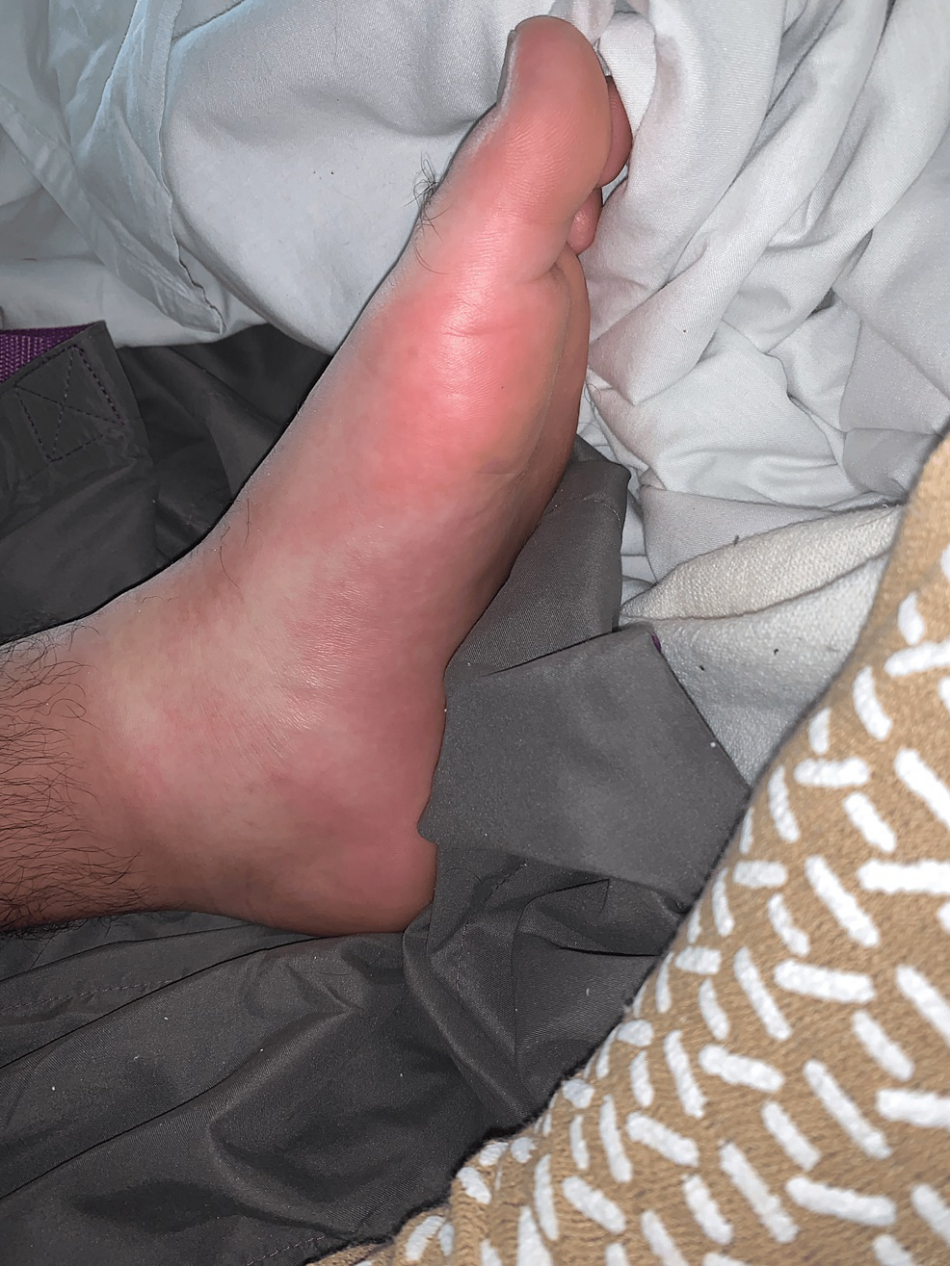
Erythema migrans rash on the patient’s left foot.

Upon physical exam, he had a blood pressure of 109/61 mmHg and a heart rate of 45 beats per minute with an irregular rhythm. An erythema migrans rash was noted on the patient’s feet (Figure 2). He described having a similar rash on his chest earlier in the month that had resolved on its own. He had an elevated white blood cell count of 10.94 (reference range 3.5-10.5).

He was admitted to the intensive care unit and cardiology was consulted due to his arrhythmia. A chest x-ray and echocardiogram were completed as part of his work-up and both were unremarkable. The decision was made to implant a temporary pacemaker to regulate his heart rhythm and prevent further episodes of syncope and hypotension while in the hospital.

Infectious disease consultation was obtained to address his symptoms as they were suggestive of a potential tick-borne illness. Empiric intravenous ceftriaxone and doxycycline were administered due to the suspicion of Lyme disease and the disseminated presentation affecting his heart. Laboratory testing was performed to confirm this diagnosis. An upper respiratory pathogen panel and serology for Rocky Mountain Spotted Fever, Ehrlichia, and Babesia were negative. He had a negative blood culture and an anti-nuclear antibody test. His erythrocyte sedimentation rate and procalcitonin were both in the normal range. His C-reactive protein was elevated at 3.7 mg/dL (reference range <0.5 mg/dL). Most importantly, his Lyme disease panel was reactive to the 23 Kd IgG band, 41 KH IgG band, 23 Kd IgM band, 39 Kd IgM band, and 41 Kd IgM band. He had a positive result on the Lyme disease antibody western blot IgM test and his Lyme antibody screen was increased at 7.32 (positive result is >1.09).

On the second day in the hospital, his heart rhythm improved to first-degree atrioventricular block as a result of the antibiotic treatment. The patient remained in the hospital for continuous monitoring and IV antibiotic administration. Over the next few days, his pacemaker settings were gradually decreased, and he was reassessed daily for atrioventricular block improvement.

There was no evidence of pacemaker dependency for 24 consecutive hours on the fifth day in the hospital; thus, the pacemaker was deactivated for a trial period. He stayed in sinus rhythm during this trial; therefore, the temporary pacemaker was safely removed. Following removal, non-sustained ventricular tachycardia and premature ventricular contractions were recorded, so a cardiac MRI was completed. It was negative for an acute etiology.

He was discharged home six days after admission. Infectious disease recommended continuation of outpatient IV antibiotics via a PICC line. He was prescribed daily ceftriaxone and doxycycline every 12 hours, both for seven days. He was asked to follow up as an outpatient with infectious disease and cardiology to monitor his recovery.

## Discussion

The incidence of Lyme disease is increasing in many countries. [4] History of an erythema migrans rash, fatigue, headache, malaise, fever, myalgia, and/or arthralgia should raise suspicion for tick-borne illness [5]. Having this suspicion for all patients presenting with these symptoms can lead to earlier treatment and decreased complications. Early Lyme disease requires treatment with doxycycline, amoxicillin, or cefuroxime for 14 days. Disseminated Lyme carditis requires IV ceftriaxone for 21-28 days and may require temporary pacing. In the event of disseminated Lyme disease, antibiotics should be started even with pending antibody titers [1]. Lyme carditis can present with atrioventricular blocks or with new bundle branch blocks. With antibiotic therapy, most patients make a full recovery; however, about 30% of patients require temporary pacing [5]. In this case, a known history of a tick bite helped to diagnose Lyme carditis quickly. The temporary pacemaker allowed for control of the patient’s heart rhythm and prevented further syncopal episodes while the antibiotics worked to decrease the infectious burden and inflammation. Eventually, the patient’s heart rhythm returned to normal, and the temporary pacemaker was no longer needed. The antibiotic therapy was continued after discharge to ensure clearance of the bacteria.

## Conclusions

It is important to keep Lyme disease on the list of differential diagnoses for any patient presenting with flu-like symptoms, even in the absence of a tick bite or travel to endemic areas. Though Lyme disease is more common in the northeast and upper Midwest of North America, there have been reports of cases all over the world. Therefore, the absence of travel history alone should not eliminate the possibility of Lyme disease. In addition, some patients may not know that they have been bitten by a tick. Ticks are small and can easily be overlooked, or they might detach from the patient before they are noticed. Though it is important to ask about the history of tick bites, a reported absence might not always be accurate. Another classic sign of Lyme disease is the erythema migrans rash, though not every case will present with it. Again, the absence of this rash alone should not eliminate the possibility of Lyme disease.

In this case, the patient’s travel history to Massachusetts, known history of a tick bite, and prominent erythema migrans rash allowed Lyme carditis to be diagnosed rapidly and allowed for prompt treatment, leading to his quick recovery. It is clear how difficult Lyme disease could be to diagnose in the absence of these unique signs. Without this classic history, other common illnesses would appear to be a more likely explanation of the flu-like symptoms. In fact, even with this known history, treatment was delayed because the initial symptoms were thought to be due to a common cold. This case report shows just how quickly Lyme disease can disseminate when untreated and is a great example of the grave consequences that can occur. It emphasizes the importance of keeping clinical suspicion for Lyme disease in all patients as this will lead to faster diagnosis, treatment, and recovery, and will prevent the serious complications that can occur.
